# Imagery practice of motor skills without conscious awareness?: a commentary to Frank et al.

**DOI:** 10.1007/s00426-023-01907-8

**Published:** 2024-01-02

**Authors:** Herbert Heuer

**Affiliations:** Leibniz Research Centre for Working Environment and Human Factors, Dortmund, Germany

## Abstract

Modifications of imagined sensory consequences will not benefit overt performance when they cannot be transformed into motor outflow that produces them. With physical practice, the acquisition of internal models of motor transformations is largely based on prediction errors that are absent in imagery practice. What can imagery practice nevertheless contribute to transformation learning? Explicit, strategic adjustments to novel transformations should be possible. This appears less likely for implicit adjustments. Are there variants of imagery practice that can produce adjustments without conscious awareness of the transformation and/or the resultant movement changes?

According to Frank et al. 2023 (this volume; also Bach et al., 2022), the benefits of imagery (or mental) practice for motor performance result from the stabilization and/or modification of imagined sensory consequences of movements. To be of any use for overt performance, however, the imagined sensory consequences must be transformed into motor outflow that produces them. The distinction between improving the desired sensory consequences on the one hand and learning to transform them into appropriate movements on the other hand is reminiscent of the distinction between trajectory (or target pattern) learning and transformation learning (Heuer & Lüttgen, 2015; cf. Heuer, 1983; Reinkensmeyer & Patton, 2009). From the perspective of the learner, learning a certain trajectory (or target pattern) of an end effector means learning the corresponding sensory consequences. Transformation learning is learning to use the desired sensory consequences for the generation of motor outflow that produces sensory consequences that match (or approximate) the desired ones. Whereas mental practice is clearly effective for learning desired sensory consequences of movements, its benefits for transformation learning are less clear.

Motor transformations are bi-directional, with the direction from motor outflow to sensory consequences being called “forward” and the direction from desired sensory consequences to motor outflow “inverse”. Here I neglect the distinction between internal models of forward and inverse transformations because, in principle, it is sufficient to acquire one type of internal model because the other type can be derived from it. In physical practice, the acquisition of an internal model of a transformation is primarily based on errors: the desired sensory consequences of a movement are mapped on some estimate of the appropriate motor outflow, and the resulting sensory consequences are compared with the predicted ones. The prediction errors, the deviations between predicted and actual sensory consequences, are considered essential training signals for the acquisition of internal models of transformations (e.g., Krakauer et al., 2019). During imagery practice, there are no prediction errors. However, when periods of imagery practice, which allow improvements of desired sensory consequences without distractions by actual movement production, alternate with periods of physical practice, which allow making use of prediction errors for transformation learning, the partial separation of learning trajectories and transformations could contribute to the often superior benefits of combined imagery and physical practice (e.g., Simonsmeier et al., 2021). But without any physical practice, how could transformations be learned or existing internal models be improved upon?

Transformation learning involves explicit (or strategic) adjustments we are consciously aware of and implicit adjustments outside conscious awareness (cf., Heuer & Sülzenbrück, 2012). For example, when we draw circles and modify the transformation of movement into the motion of a cursor on a monitor, corrections can be unintended and unnoticed by the performer or intended and strategic, and these two types of corrections are additive (Sülzenbrück & Heuer, 2009). More popular than circle drawing is a task where participants perform movements in different directions and the direction of visual feedback provided by a cursor on a monitor is rotated relative to the direction of hand movement. With this task, Mazzoni and Krakauer (2006) also found essentially additive strategic and implicit adjustments. These adjustments even over-compensated the rotation, leading to an error in the opposite direction which could be reduced in the long run (e.g., Rand & Rentsch, 2015). By now the distinction between explicit and implicit processes involved in learning transformations (mostly visuo-motor rotations) is widely accepted (e.g., Heuer & Hegele, 2008; Maresch et al., 2020; Taylor et al., 2014).

Explicit or strategic adjustments to a transformation can certainly be improved by imagery practice. For example, movement errors can be imagined and shape motor behavior, at least when the imagined errors are systematic (e.g., Powell, 1973). Rotations of visual feedback can be instructed, and such instructions can be used for strategic corrections of pointing movements (e.g., Hegele & Heuer, 2010), most likely also for imagery practice of such corrections.

What about implicit adjustments to transformations? Movements can be adjusted to visuo-motor rotations up to about 30° without participants noticing the rotation (Werner et al., 2015), in particular when the rotation is introduced in successive small steps (Kagerer et al., 1997). Adjustments of movement directions which remain unnoticed can also be induced when visual feedback has a constant rotation relative to the target rather than relative to the hand movement and participants are instructed to ignore it (Morehead et al., 2017). Adjustments can even be induced without overt movements when a stop signal is presented after a preparatory phase, followed by rotated fake visual feedback (Kim et al., 2022). From these findings, it is only a small step to questions about possible effects of imagery practice. Can imagery practice induce adjustments of motor behavior to modified transformations we are not aware of? For example, will the directions of overt movements in a subsequent test be modified after a practice period with imagined movements and fake visual feedback that is slightly displaced from a previously presented target, even when this displacement remains unnoticed?

An apparent instance of learning a transformation by imagery practice without awareness of the transformation has been reported by Michel et al. (2013), followed up by Fleury et al. (2023). Both studies showed prism adaptation: participants looked through 15° or 10° wedge prisms at a target and imagined movements to that target. None of the participants reported awareness of an intermodal conflict. Nevertheless, there was an aftereffect, although somewhat smaller than after physical practice (in the study of Fleury et al. only for participants with high motor imagery ability). At first glance, this appears as learning of a modified visuo-motor transformation by imagery practice and without conscious awareness of the modification. However, Michel et al. suggest an explanation in terms of a modified proprioceptively sensed start position of the hand during the post-test rather than a modified internal model of the visuo-motor transformation. This modification was a consequence of the intermodal discrepancy (visual and proprioceptive) during imagery practice where the prismatically displaced hand could be seen at the start position.

There are situations where internal models of transformations must be updated for accurate movements even without artificial modifications of “natural” transformations. For example, when we throw a ball or a dart, our body must be properly oriented relative to the environment because the goal is defined in extrinsic (allocentric), but motor outflow in intrinsic (egocentric) frames of reference. The transformation of the desired sensory consequences of throwing into appropriate motor outflow requires short-term adjustments (or calibration) of the internal model because the transformation is changing on a short time scale—there are slight variations of posture in relation to the target of the throws. Such rapid adjustments can be evidenced in improvements across the series of three darts in professional players (Wunderlich et al., 2020) or three free throws of NBA basketball players (Phatak et al., 2020). Would such subtle adjustments also be observed with imagined throws? Would a physical dart after two imagined ones have the accuracy of a third physical dart or only that of a first one? My bet would be the latter.

In conclusion, whereas with physical practice implicit adjustments to modified (visuo-motor) transformations are well established, such adjustments without conscious awareness of the modification could be out of reach of imagery practice. Of course, this conclusion is tentative and can be dismissed by evidence to the contrary.

## Data Availability

Not applicable.
